# Exploring the molecular mechanism associated with breast cancer bone metastasis using bioinformatic analysis and microarray genetic interaction network

**DOI:** 10.1097/MD.0000000000012032

**Published:** 2018-09-14

**Authors:** Xinhua Chen, Zhe Pei, Hao Peng, Zhihong Zheng

**Affiliations:** a Department of Medical Oncology, Fujian Cancer Hospital & Fujian Medical University Cancer Hospital, Fuzhou, Fujian, China; b Duke University Medical School, Durham, NC; c Guangxi Key Laboratory of Veterinary Biotechnology, Guangxi, China; d Department of Hematology, Fujian Provincial Key Laboratory of Hematology, Fujian Medical University Union Hospital, Fuzhou, Fujian, China.

**Keywords:** bone metastasis, breast cancer, enrichment analysis, interaction network, microarray

## Abstract

**Background:**

Bone metastases are common in advanced breast cancer patients and frequently leading to skeletal-related morbidity and deterioration in the quality of life. Although chemotherapy and hormone therapy are able to control the symptoms caused by bone destruction, the underlying molecular mechanisms for the affinity of breast cancer cells towards skeletal bones are still not completely understood.

**Methods:**

In this study, bioinformatic analysis was performed on patients’ microarray gene expression data to explore the molecular mechanism associated with breast cancer bone metastasis. Microarray gene expression profile regarding patients with breast cancer and disseminated tumor cells was downloaded from Gene Expression Omnibus (GEO) database (NCBI, NIH). Raw data were normalized and differently expressed genes were identified by using Significance Analysis of Microarrays (SAM) methods. Protein interaction networks were expanded using String. Moreover, molecular functions, biological processes and signaling pathway enrichment analysis were performed using Gene Ontology (GO) and Kyoto Encyclopedia of Genes and Genomes (KEGG).

**Results:**

We identified 66 differentially expressed genes. After submitting the set of genes to String, genetic interaction network was expanded, which consisted of 110 nodes and 869 edges. Pathway enrichment analysis suggested that adhesion kinase, ECM-receptor interaction, calcium signaling, Wnt pathways, and PI3K/AKT signaling pathway are highly associated with breast cancer bone metastasis.

**Conclusion:**

In this study, we established a microarray genetic interaction network associated with breast cancer bone metastasis. This information provides some potential molecular therapeutic targets for breast cancer initiation and progression.

## 1 Introduction

Globally, breast cancer is the most frequently diagnosed cancer and the leading cause of cancer-related death in women.^[1]^ Metastatic diseases occur in most women with advanced breast cancer and bone is one of the most preferential distant organs for metastasis of breast cancer. Evidence from clinical and postmortem studies suggests that 47% to 85% of breast cancer patients will have bone metastasis.^[2]^ It has also been reported that the breast cancer tumor subtypes affect the metastases sites and rates. The lowest rate of bone metastases are patients with estrogen (ER)-negative/human epidermal growth factor receptor 2 (HER2)-negative tumors, which is 55.2%; meanwhile, this rate was significantly increased to 69.8% (HER2-positive tumors), 87.8% (ER-positive/HER2-negative/Ki67high tumors), and 73.1% (ER-positive/HER2-negative/Ki67low tumors). The most common sites of bone metastases are the spine, ribs, pelvis, proximal femur, and skull. The destruction of these bones frequently leads to excessive skeletal-related complication such as bone pain, pathological fractures, life-threatening hypercalcemia, spinal cord compression, and other nerve compression syndromes. Some of them can be fatal and significantly reduce the quality of life.

Bone metastasis is a complex, multistage process that requires breast cancer cells to detach from the primary tumor, travel through the blood or lymphatic system, survive in bone microenvironment, and then proliferate in bone tissue.^[3]^ To date, genomic studies have suggested that each step of metastasis was associated with a series of molecular events. However, the interaction network of molecular mechanism associated bone metastases from breast cancer is still not completely understood. Motivated by this, we established a comprehensive protein interaction network by building a microarray gene expression profile originating from breast cancer patients with bone metastases, hoping to reveal the molecular mechanisms in breast cancer bone metastasis. In our analysis, 66 genes with significant expression changes were identified to confer bone metastasis. Pathway enrichment analysis highlighted that adhesion kinase, extracellular matrix (ECM)–receptor interaction, calcium signaling pathway, and phosphatidylinositol 3-kinase (PI3K)/protein kinase B (AKT) signaling pathway are potential key regulators, which may involve in breast cancer bone metastasis. These results advanced our understanding of molecular information of bone metastasis from breast cancer and provided potential targets for clinical interventions.

## 2 Material and methods

### 2.1 Microarray dataset resources

After searching in Gene Expression Omnibus (GEO, http://www.ncbi.nlm.nih.gov/geo/), a public functional genomics data repository, a microarray dataset was downloaded with the accession number GSE14776. In this study, Cawthorn et al^[4]^ explored the analyzable yield of genetic material from human biopsy samples in order to describe differences in gene expression between disseminated tumor cells and bone metastatic tumor cells. Total RNA was extracted from disseminated tumor cells and bone metastatic tumor cells and mRNA array was performed on Illumina HumanRef-8 v3.0 platform. Other involved online databases were listed in the String website.

### 2.2 Aberrant expressed genes identification

To standardize the microarray data set, comparison of the gene expression profiles of metastatic tumor cells versus disseminated tumor cells was normalized using log2 transformation, a method previously developed by Fan et al.^[5]^ Subsequently, Significance Analysis of Microarrays (SAM, http://statweb.stanford.edu/∼tibs/SAM/) was applied to produce a cluster of up- or downregulated variant genes according to previous publications.^[6,7]^

### 2.3 Functional protein association network construction

Protein–protein/Gene–protein interaction networks were expanded on the basis of the result from 2.2 using String consortium (http://string-db.org/).^[8]^ Gene Ontology consortium (GO, http://www.geneontology.org/) and Kyoto Encyclopedia of Genes and Genomes (KEGG, http://www.genome.jp/kegg/) functional enrichment were also applied via Database for Annotation, Visualization and Integrated Discovery (DAVID, https://david.ncifcrf.gov/).^[9,10]^

### 2.4 Statistical analysis

Gene expression was considered to be significant if the threshold of false discovery rate (FDR) ≤5% and fold change ≥5. For GO and KEGG enrichment analysis, biological process, molecular function, and signaling pathways were identified as different if the *P* value was ≤5%.

### 2.5 Ethical Experimentation

The study does not involve any patient consent, so ethical approval is not necessary.

## 3 Results

### 3.1 Sixty-six genes were found to be significantly expressed in bone-specific metastatic breast tumor cells

**Figure 1:**
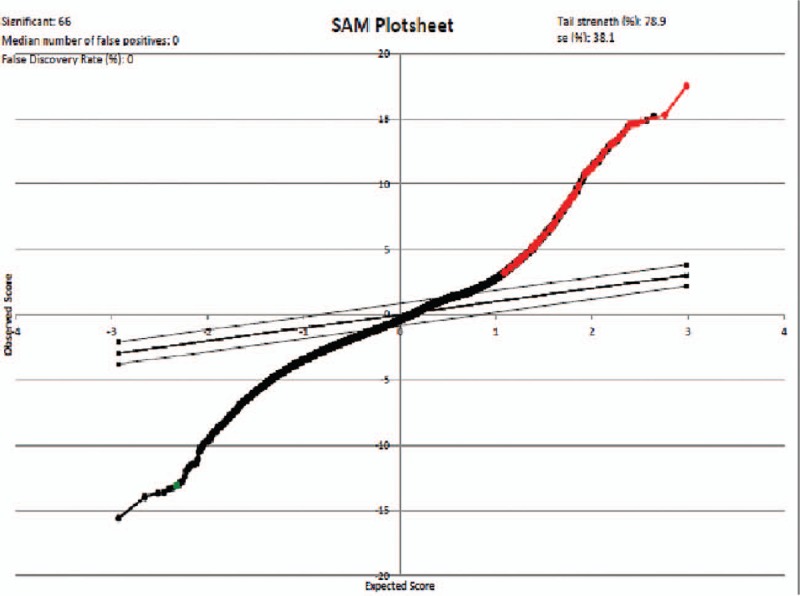
Significance analysis of microarrays (SAM) plot result generated by SAM plugin in Excel platform.

A total of 14 breast tumor samples were profiled in this study, consisting of 8 disseminated tumor cell samples and 6 metastatic tumor cell samples. After performing SAM, 66 genes were found to be differently expressed in metastatic tumor cells comparing to disseminated tumor cells as shown in Fig. 1 and Table 1. Totally, 65 genes increased and 1 gene decreased dramatically with the threshold of FDR ≤5% and fold change ≥5.

**Table 1:**
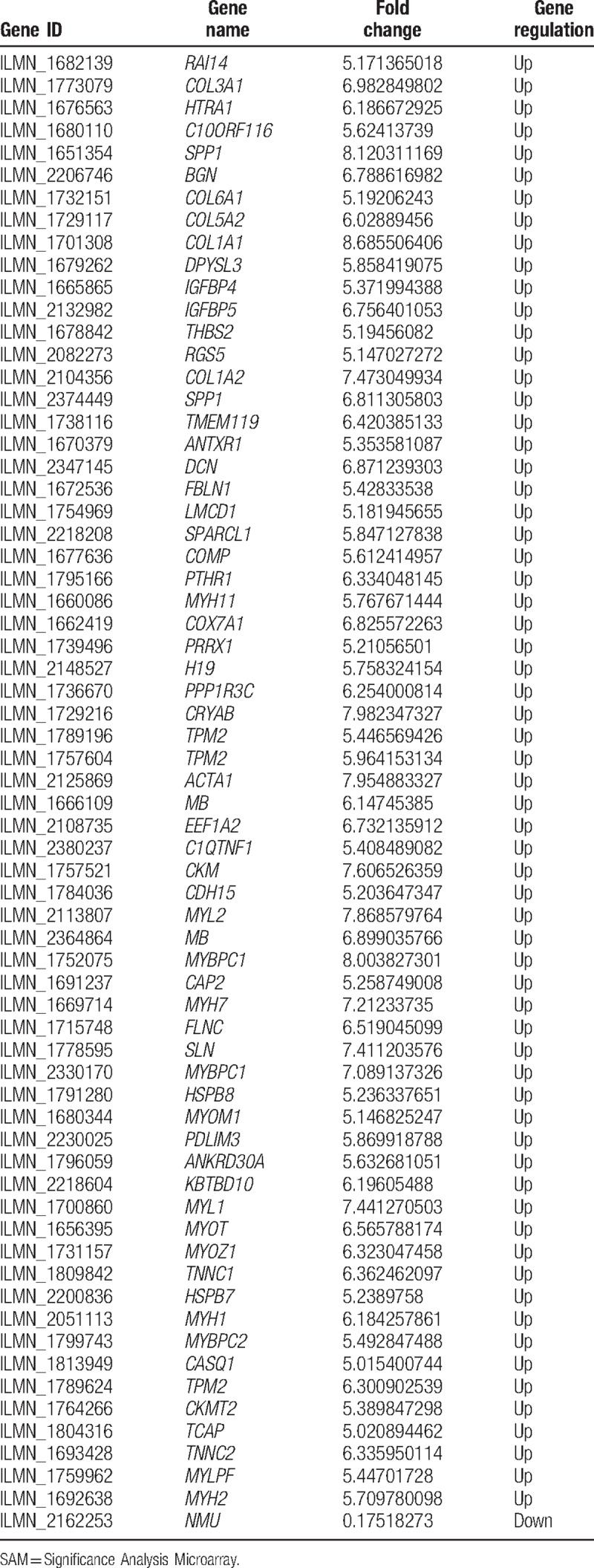
Significant genes identified by significant analysis of microarray (SAM) in metastatic tumor cells versus disseminated tumor cells.

### 3.2 Gene–gene interaction network construction associated with breast cancer bone metastasis

**Figure 2:**
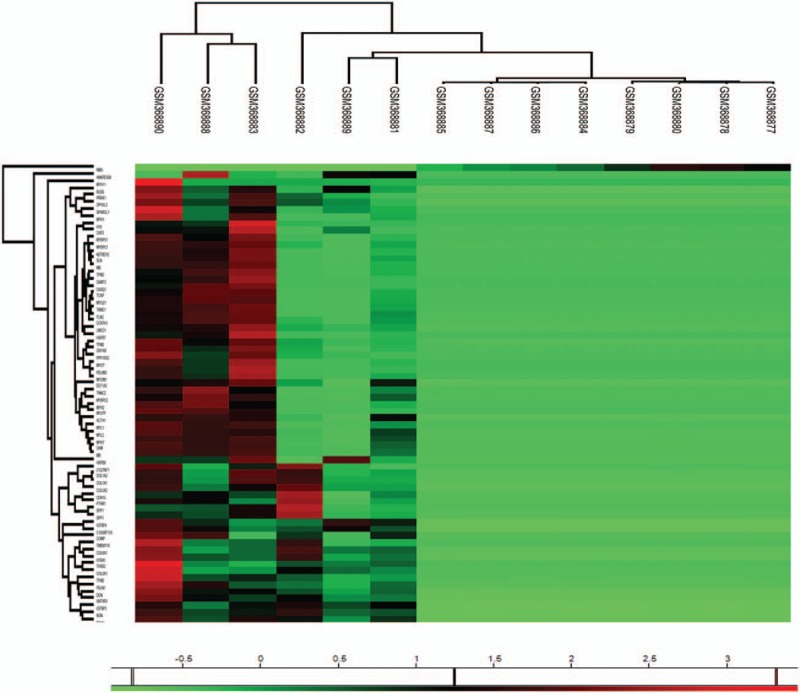
Heatmap visualization of the significant genes identified by SAM. All the detailed information can be seen in Table 1.

**Figure 3:**
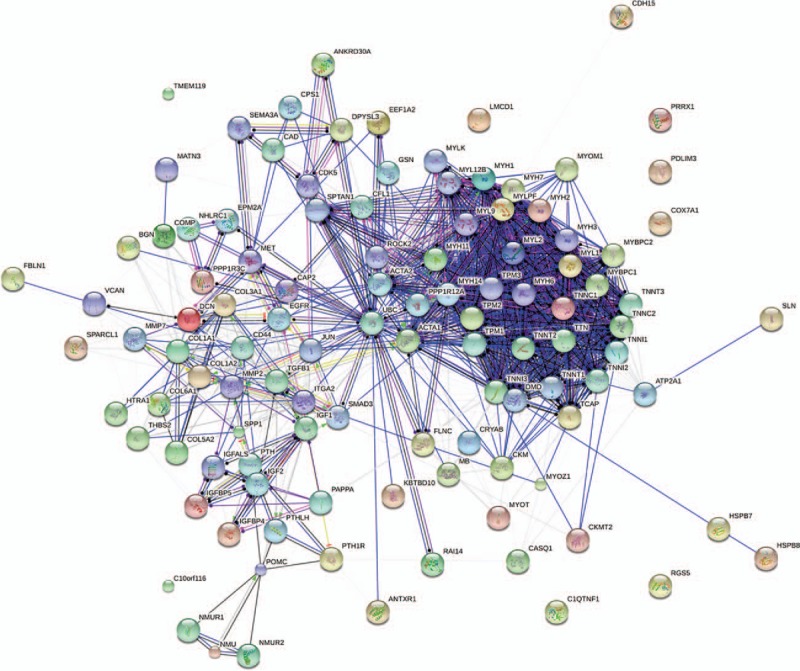
Gene to gene interaction network associated with breast cancer bone metastasis generating by String platform.

To better identify how these genes regulated breast cancer bone metastasis in a system biology perspective, all these significant genes were applied to String platform for further analysis. As shown in Fig. 2, the interaction network involved in bone metastasis consists of 110 nodes and 869 edges with the average node degree of 15.8. Network analysis also indicated that the clustering coefficient (cc) was 0.58, which means that the network has a reliable robustness Figure 3.

### 3.3 GO analysis in terms of molecular function and biological processes

To explore the genetic interaction network involved in bone metastasis in the context of GO, all the nodes were submitted to DAVID for functional annotation. As summarized in Table 2, molecular function analysis indicated that most of these genes regulated protein binding and activities. We also elevated the biological processes involved in this bone metastasis network (Table 3). Table 3 summarized all the potential biological processes for bone metastasis. In particular, all these genes seemed to be involved in skeletal muscle development and differentiation, and cell development Table 4.

**Table 2:**
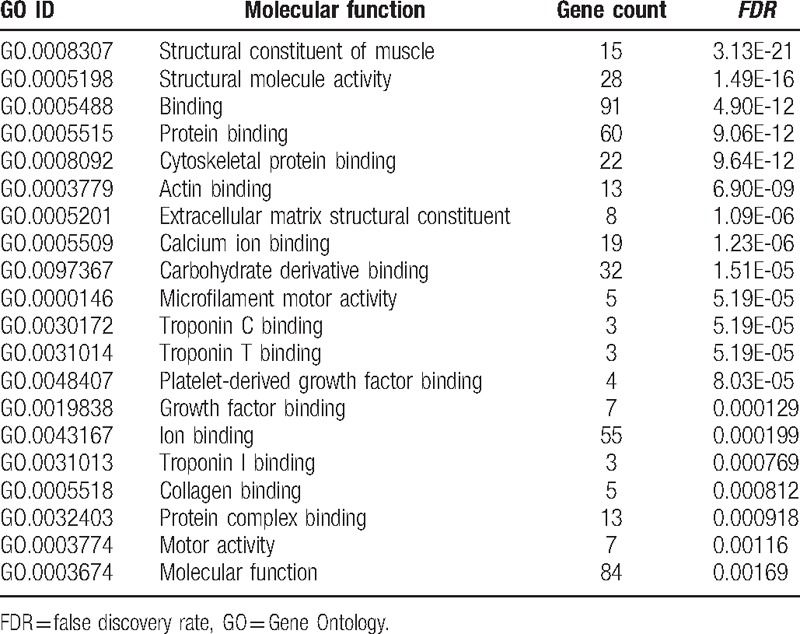
Molecular function analysis of the genetic interaction network associated with metastatic tumor cells in terms of Gene Ontology (GO).

**Table 3:**
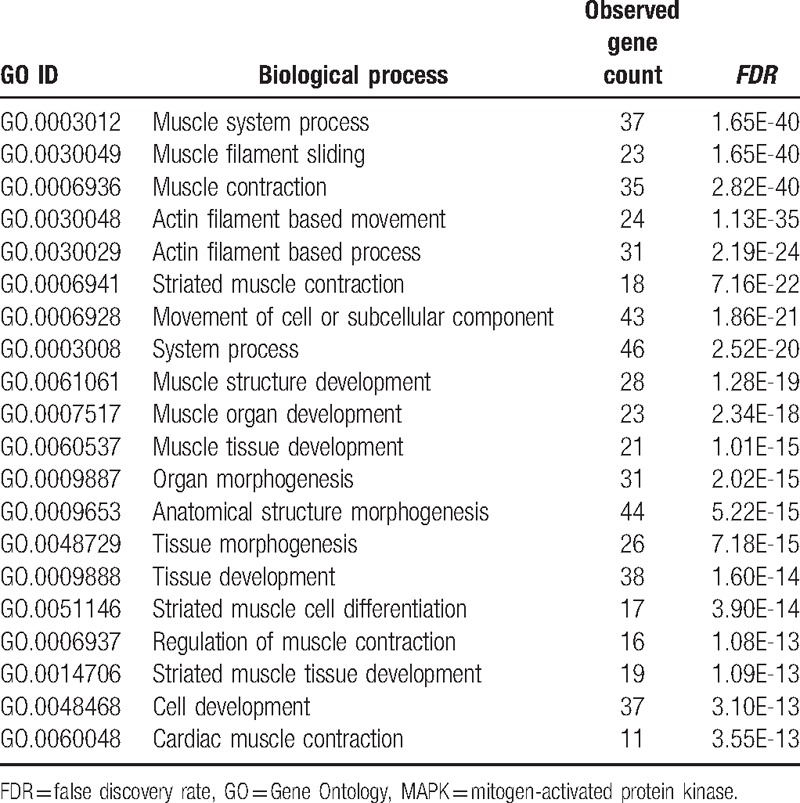
Biological process analysis of the genetic interaction network associated with metastatic tumor cells in terms of Gene Ontology (GO).

**Table 4:**
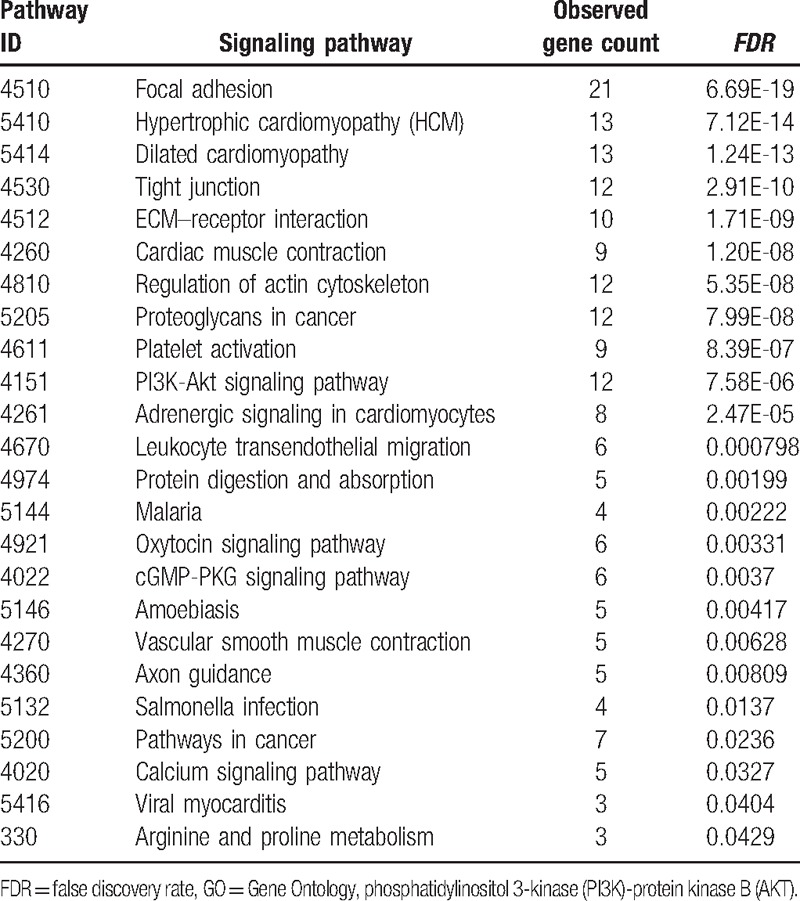
Signaling pathway analysis of the genetic interaction network associated with metastatic bone cells in terms of Gene Ontology (GO).

### 3.4 Signaling pathway enrichment analysis

To assess the relationship between the significantly expressed genes and bone metastasis, we also elevated the potential signaling pathways involved in this pathogenesis (Table 3). Notably, focal adhesion kinase (FAK), ECM–receptor interaction, calcium signaling pathway, and PI3K/AKT signaling pathways seem to confer bone metastasis in metastatic tumor cells.

## 4 Discussion

As we described previously, breast cancer bone metastasis is a complex process that includes tumor cells dissemination into circulation, homing to bone, and proliferation in bone tissue. Underlying these complicated, multistep scenarios, it has been known that a sophisticated network of molecular events is crucial in the development of metastasis to bone, which was not fully understood. In this literature, the authors identified a microarray gene expression profile and established a comprehensive genetic interaction network to reveal the molecular mechanisms in breast cancer bone metastasis. The results suggested that ECM–receptor interaction, FAK, calcium signaling pathway, and PI3K/AKT signaling pathway were highly associated with breast cancer bone metastasis.

Previous publications have already confirmed the role of ECM components in breast cancer dissemination and metastases.^[11,12]^ As polysaccharides and fibrous proteins, ECM which induced by either cancer cells or stromal components is a crucial component of cancer microenvironment, initiating downstream signaling events that lead to the aggressive behavior of breast cancer.^[13,14]^ The interaction of cancer cells and ECM components is profoundly altered at all steps of cancer metastasis, which include detachment from the primary tumor, migration through adjacent tissue, invasion into and extravasation from the vasculature.^[15–17]^ Studies on interaction of tumor cells with ECM components showed increased extracellular protease activity mediated by the family of matrix metalloproteinases (MMPs).^[18,19]^

Several previously studies indicated that FAK mediated cancer metastasis in various cancers.^[20–22]^ (FAK) is a nonreceptor protein tyrosine kinase that resides at the sites of at focal adhesions, which plays an essential role in cancer cells survival, proliferation, migration, and invasion.^[23,24]^ FAK coordinates a signaling network that orchestrates these processes through both kinase-dependent and independent mechanisms.^[25]^ FAK cooperates with SRC and leads to SRC phosphorylation and then FAK/SRC phosphorylation at multiple sites, relaying the external signal into cells associated with various genes and multiple signaling pathways, such as PI3K/AKT and MAPK.^[26]^

A previous study suggested the regulation of the metastasis formation either directly through mutations in the involved adhesion molecules or indirectly through impaired calcium signaling pathway.^[27]^ The ubiquitous second messenger calcium is one of the crucial regulators that will be involved in several fundamental physiological functions, such as cell cycle control, survival, and cancer metastasis.^[28,29]^ In multiple cancer metastasis stages, calcium signaling and cell adhesion interact in various ways with each other. E-cadherin, a calcium-dependent cell–cell adhesion molecule, is a major suppressor of metastasis, whose downregulation or inactivation in carcinomas has been reported to result in reduced cell adhesion, and essentially requires Ca^2+^-ions to form hemophilic interactions between 2 neighboring cells in adherens junctions.^[30,31]^ Evidences suggest that Rap2B is an upstream target of the Ca^2+^-related ERK1/2 signaling pathway in cancer cells, contributing to important events during tumor progression, such as cell proliferation, migration, invasion, and metastasis,^[32–35]^ which further attested our bioinformatic prediction.

The PI3K/AKT/mTOR pathway had been known to control many cellular functions such as proliferation, growth, survival, motility, and metabolism and proved to be related with cancer metastasis.^[36,37]^ By stimulating the expressions of Receptor activator of nuclear factor kappa-B ligand (RANKL), parathyroid hormone-related protein (PTHrP), and bone morphogenetic protein 2 (BMP-2) partly through NF-kB, PI3K/AKT pathway had been proved to play an important role in prostate carcinoma bone metastasis.^[38]^ Through the PI3K/AKT pathway, mPRα promoted the expression of MMP-9 during breast cancer cells invasion to local lymph nodes.^[39]^ The angiogenesis and metastasis of breast cancer cells were inhibited by downregulating PI3K/AKT/ERK signaling pathway mediated by connective tissue growth factor.^[40]^ Several drugs against PI3K, Mammalian target of rapamycin (mTOR), and AKT had already been invented and tested in clinical trials.

Besides the signaling pathways mentioned above, we also discovered many pathways, including tight junction, regulation of actin cytoskeleton, leukocyte transendothelial migration, etc, were involved in breast cancer bone metastasis. However, detailed information regarding the association between these pathways and bone metastasis has not been fully investigated.

In conclusion, using the integrated microarray gene expression profile and genetic interaction network, we characterized some molecular signaling pathways (ECM-receptor interaction, FAK, calcium signaling pathway, and PI3K/AKT signaling pathway), which may mediate the aggressive behavior of breast cancer in terms of bone polarization.

## Acknowledgment

We thank GEO, SAM, and String databases for making their data readily available to the scientific community.

## Author contributions

All authors contributed toward data analysis, drafting and revising the paper, and agree to be accountable for all aspects of the work.

**Conceptualization:** Zhi-hong Zheng.

**Data curation:** Xin-hua Chen.

**Formal analysis:** Zhe Pei, Hao Peng.

**Methodology:** Xin-hua Chen, Zhe Pei.

**Project administration:** Xin-hua Chen.

**Resources:** Xin-hua Chen, Zhi-hong Zheng, Zhe Pei.

**Software:** Xin-hua Chen, Hao Peng.

**Supervision:** Zhi-hong Zheng.

**Writing – review & editing:** Zhe Pei, Hao Peng.
